# Bone Morphogenetic Protein-7 (BMP-7) Promotes Neuronal Differentiation of Bone Marrow Mesenchymal Stem Cells (BMSCs) *In Vitro*

**DOI:** 10.1155/2021/7239783

**Published:** 2021-01-27

**Authors:** Heng Zhang, Wen Zhang, Guangchao Bai, Lei Gao, Kuanxin Li

**Affiliations:** ^1^Department of Orthopaedics, The First Affiliated Hospital of Bengbu Medical College, Laboratory of Tissue and Transplant in Anhui Province, Bengbu Medical College, Bengbu City, China; ^2^Department of Orthopaedics, The Second Affiliated Hospital of Medical College, Shihezi University, Xinjiang, China

## Abstract

This study is aimed at investigating the effects of bone morphogenetic protein-7 (BMP-7) on the differentiation of bone marrow mesenchymal stem cells (BMSCs) into neuron-like cells *in vitro*. The rat BMSCs were isolated and identified, which were divided into the control, empty, recombinant rhBMP-7 transfection, and Lv-BMP-7 transfection groups. BMSCs were induced under different conditions. CCK-8 assay was performed to detect cell proliferation. ALP was used to detect cell activity. Cellular morphology after induction was observed. Immunofluorescence was conducted to detect the expression and location of nerve cell markers. Quantitative real-time PCR and Western blot analysis were performed to detect the mRNA and protein expression levels, respectively. The rhBMP-7 and Lv-BMP-7 promoted the proliferation of BMSCs, accompanied with increased ALP activities. Morphological observations revealed that rhBMP-7 and Lv-BMP-7 induced BMSCs to differentiate into neuron-like cells. Immunofluorescence revealed that the rhBMP-7 and Lv-BMP-7 groups showed positive expression of MAP-2 and Nfh in BMSCs. MAP-2 was mainly distributed in the cell body and cellular protrusion, while Nfh was mainly distributed in the cytoplasm and cell protrusion. Positive mRNA and protein expressions of MAP-2 and Nfh were observed in the cells of the rhBMP-7 and Lv-BMP-7 groups, and the expression levels were significantly higher than the control and empty groups. Both exogenous BMP-7 (rhBMP-7) and endogenous BMP-7 (Lv-BMP-7) can induce BMSCs to differentiate into neuron-like cells highly expressing the neuronal markers MAP-2 and Nfh.

## 1. Introduction

Spinal cord injury (SCI) is caused by direct physical damage to the spinal cord, which causes severe neurological dysfunction [1]. Traditional medicine and surgical treatment can only reduce the edema of the damaged part of the spinal cord and restore spinal stability, and, however, due to lacking the regenerative ability of the damaged neurons, it is difficult to achieve satisfactory treatment outcomes for SCI in a clinic. At present, the researches concerning the treatment of SCI have been mainly focused on cell transplantation after SCI. Successfully transplanted neuronal cells could not only supplement the neuronal loss in the damaged area but also enhance the subsequent neurotrophic factor secretion, improving the microenvironment [2, 3]. Mesenchymal stem cells (MSCs) are characterized by self-renewal and have multidirectional differentiation potentials. Initial researches have mainly focused on transplanting MSC into the SCI animal model to replace the nerve cell loss within the lesions. A large number of studies have confirmed that the transplantation could improve the function of damaged nerves [4–7].

The bone morphogenetic protein (BMP) ligands and their receptors are abundantly expressed in the embryonic central nervous system during neurogenesis, which help to regulate cell proliferation, survival, differentiation, and apoptosis. In addition, the bone morphogenetic proteins, including BMP-7, can be reexpressed after injuries, which participate in the functional recovery of the central nervous system [8–10], which represents a signaling molecule guiding the axon regeneration during the regeneration and neuronal cell repairing. Heggeness et al. [11] have administered exogenous rhBMP-2 (60 ng/10 *μ*l each mouse) after mechanical injuries of the sciatic nerve in mice and shown that the sciatic nerve cells have obvious morphological changes, and the nerve cells rapidly proliferate and migrate to the surrounding tissues. The BMP signal has been considered to be the main way to promote the synaptic growth of neurons. Berke et al. [12–14] have shown that the addition of BMP would trigger multiple processes of neuron expansion and expression of dendritic morphology, as well as the cytoskeleton and ultrastructural features. In the rat SCI model, Setoguchi et al. [15] have shown that the expression levels of BMP-7 in the spinal cord tissue are increased in the early stage of SCI, indicating that BMP-7 plays a protective role in the early injury stage. A large number of studies have confirmed that BMP plays an important role in neuron regeneration, and BMP-7 has a repairing effect on nerve damages [17–21]. A previous study from our lab has also confirmed that, after the administration of recombinant BMP-7 into the spinal arachnoid cavity of the SCI model rats, the motor function of rats would be restored [16]. However, the role of BMP, including BMP-7, in the differentiation of BMSCs to neuron-like cells has not been reported.

Based on the above findings, we hypothesized that BMP-7 would induce BMSCs to differentiate into nerve cells, participating in the repairing process of SCI. Therefore, in this study, the bone marrow mesenchymal stem cells (BMSCs) were isolated, which were subjected to the exogenous rhBMP-7 intervention and BMP-7 lentivirus transfection. The effects of BMP-7 on the growth and differentiation of BMSCs were monitored, to provide basic research data for the repairing and treatment of SCI in a clinic.

## 2. Subjects and Methods

### 2.1. Isolation and Culture of BMSCs

Totally 10 male SD rats, 4-week-old, weighing 120 ± 27 g, were purchased from the Experimental Animal Center of Xinjiang Medical University (authorization No. SCXK (Xinjiang) 2015-001). Rats are housed in an animal room, in a 12-hour light/dark cycle (lit at 08:00), as well as normal temperature (25 ± 1°C) and humidity of 60 ± 10%, with free access to food and water. Animal experimental procedures were performed in compliance with the Guide for the Care and Use of Laboratory Animals of the National Research Council of the Nations (revised 2010-12) and approved by the Ethics Committee of Xinjiang Production and Construction Corps Hospital, China.

For the BMSC isolation and culture, the rats were sacrificed by cervical dislocation, and the femurs and tibias were removed. Under sterile conditions, the bone marrow was washed out from the femur and tibia, with the DMEM complete medium (Cat# 11995065, Gibco, Waltham, Massachusetts, USA) containing 10% fetal bovine serum (Cat# 30084.03, FBS; HyClone, Logan, Utah, USA), supplemented with 1% penicillin-streptomycin (100 U/ml; Cat# SV30010.01, HyClone, Logan, Utah, USA). After centrifugation at 300 ×g for 7 min, the supernatant was discarded, and the cells were suspended with the DMEM complete medium to obtain the single-cell suspension. The cells were seeded onto the T-25 cell culture flask, at a density of 1 × 10^8^ cells/ml, which were cultured in a 5% CO_2_, 37°C incubator for 48 h. The nonadherent cells were removed, and the adherent cells were cultured with the fresh DMEM complete medium. The culture medium was changed every three days.

### 2.2. Flow Cytometry Analysis

The BMSCs of the second passage were collected, which were washed with PBS and subjected to centrifugation at 300 ×g for 7 min. Then, the cells were resuspended and totally 100 *μ*l cells (1 × 10^7^ cells/ml) were added into the detection tube, which were incubated with 1 *μ*l FITC-conjugated anti-rat CD44 antibody (0.5 mg/ml; 203906-FITC; BioLegend, San Diego, CA, USA), as well as 1 *μ*l PE-conjugated anti-mouse/rat CD29 antibody (0.2 mg/ml; 102207-PE; BioLegend) in the dark at 4°C for 20 min. After washing with PBS, the cells were resuspended with 500 *μ*l PBS, which were subjected to the detection with a flow cytometer (E6; Mindray, Shenzhen, Guangdong, China). For each sample, a total of 20,000 particles in the target cell population were recorded. The detection data were analyzed with the FlowJo™ software (V7.6; BD).

### 2.3. Lentiviral Vector-BMP-7 (Lv-BMP-7) Transfection and Grouping

The Lv-BMP-7 and corresponding vehicle control (Ubi-MCS-3FLAG-CBh-gcGFP-IRES-puromycin) were purchased from GeneChem (Shanghai, China). According to the manufacturer's instructions, the appropriate multiplicity of infection (MOI) was determined to evaluate the optimal virus titer for lentivirus transfection, and the transfection efficiency was observed with the fluorescence inverted microscope.

For the experimental grouping, BMSCs from the second passages were divided into the following four groups: (1) the control group, in which the cells were cultured with the DMEM complete medium; (2) the lentiviral vector control group, in which the cells were transfected with the empty lentiviral vector for 72 h; (3) the rhBMP-7 group, in which the cells were treated with the recombinant rhBMP-7 (75 ng/ml; 120-03P-100; PeproTech) for 2 h; and (4) the Lv-BMP-7 group, in which the BMSCs were transfected with the Lv-BMP-7 for 72 h.

### 2.4. Cell Viability Assessment

The cells were seeded onto the 96-well plates, at a density of 1 × 10^5^ cells/ml, in 100 *μ*L culture medium each well. After being cultured in a 37°C, 5%CO_2_ incubator for 24 h, the cells were subjected to different treatments, respectively, for 7 days. Then, 10 *μ*l CCK-8 solution (CA1210; Solarbio, Wuhan, Hubei, China) was added into each well to incubate the cells at 37°C for 4 h. The absorbance at 450 nm was added with the Multiskan GO microplate reader (Thermo, Waltham, Massachusetts, USA). Each group had 6 replicate wells, and the cell viabilities were calculated accordingly.

### 2.5. Alkaline Phosphatase (ALP) Activity Assessment

At indicated time points, the cells were collected and washed with PBS, followed by centrifugation at 300 ×g for 7 min. The cells were lysed with 0.2% Triton X-100 lysis. After centrifugation at 300 ×g for 7 min, the supernatant was discarded. Reaction solution within the ALP assay kit (Beyotime) was added into the precipitation and incubates the cells at 37°C for 30 min. The absorbance at 450 nm was added with the Multiskan GO microplate reader (Thermo) to determine the ALP activities. Each group had 6 replicate wells, and the ALP activity standard curve was calculated and obtained accordingly.

### 2.6. Cellular Immunofluorescence

The BMSCs were seeded onto the 96-well plates, at a density of 1 × 10^5^ cells/ml, in 100 *μ*L culture medium each well. After intervention under different conditions, the medium was aspirated and the cells were gently washed with PBS 3 times. A total of 30 *μ*l paraformaldehyde (4%) was added to each well to fix the cells for 15 min, followed by another round of washing with PBS 3 times. After being treated with 0.5% Triton X-100 at room temperature for 20 min and the following washing, the cells were blocked with 5% BSA solution at room temperature for 20 min. Then, the cells were incubated with the rabbit anti-Nfh primary antibody (1 : 1500 dilution; bs-10680R; Bioss), or rabbit anti-MAP-2 primary antibody (1 : 1500 dilution; bs-1369R; Bioss), at 4°C overnight. After washing, the cells were incubated with fluorescent secondary antibody (1 : 500 dilution; bs-0295G-FITC; Bioss) at 37°C in the dark for 2 h. After washing, the cells were incubated with DAPI (C0060; Solarbio) in the dark for 5 min. Fluorescence was observed with a fluorescence inverted microscope (Axio Observer A1, Zeiss, Germany), and the images were taken and analyzed.

### 2.7. Quantitative Real-Time PCR

Cells were collected, and total RNA was extracted with the extraction kit (DP431; Tiangen, Beijing, China). The cDNA was obtained from the RNA with the PrimeScript TM RT kit (Cat. No., RR037A; Takara Bio, Inc.). The quantitative real-time PCR was performed on the AGS PCR machine (AFD4800; Anyu Technology Co., Ltd., Hangzhou, Zhejiang, China). The primer sequences were as follows: Nfh, forward 5′-GATGGCATTGGACATTGAGA-3′ and reverse 5′-GAGAGTAGCCGCTGGTTATG-3′; MAP-2, forward 5′-GGACATCAGCCTCACTCACA-3′ and reverse 5′-CCTTCCTCCTCCTCTCTGTATG-3′; and GAPDH, forward 5′-CTCTCTGCTCCTCCCTGTTC-3′ and reverse 5′-GCCAAATCCGTTCACACCG-3′. The reaction conditions were set as follows: 95°C for 10 s; 95°C for 10 s, for a total of 40 cycles; and 60°C for 34 s. The relative mRNA expression levels of target genes were calculated using the 2^-*ΔΔ*Ct^ method. GAPDH was used as internal reference.

### 2.8. Western Blot Analysis

Cells were lysed with the protein lysis buffer (BL504A; Biosharp). Protein concentration was determined with the BCA method (BL521A; Biosharp). A total of 40 *μ*g protein sample was separated by 10% SDS-PAGE, which was then electronically transferred onto the PVDF membrane (0.45 *μ*m; Millipore). After blocking with 5% BSA at room temperature for 1 h, the membrane was incubated with the rabbit anti-NF-H primary antibody (1 : 1500 dilution; bs-10680R; Bioss), or rabbit anti-MAP-2 primary antibody (1 : 1500 dilution; bs-1369R; Bioss), at 4°C overnight. After washing, the membrane was incubated with the goat anti-rabbit IgG/HRP secondary antibody (1 : 5000 dilution; bs-0295G-HRP; Bioss) at room temperature for 1 h. Color development was performed with the ECL method (PE0010; Solarbio). The protein bands were imaged and analyzed with the ImageJ software (V1.52s; Bharti Airtel Ltd., Co.). GAPDH was used as internal reference.

### 2.9. Statistical Analysis

Data were expressed as mean ± SEM. Statistical analysis was performed with the SPSS v19.0 software (v19.0; IBM). One-tailed *t*-test was conducted for the pair-wise comparison, while one-way ANOVA was performed for the multiple group comparison, with the Tukey posttest. *P* < 0.05 was considered statistically significant.

## 3. Results

### 3.1. Culture, Identification, and Transfection of BMSCs

**Figure 1:**
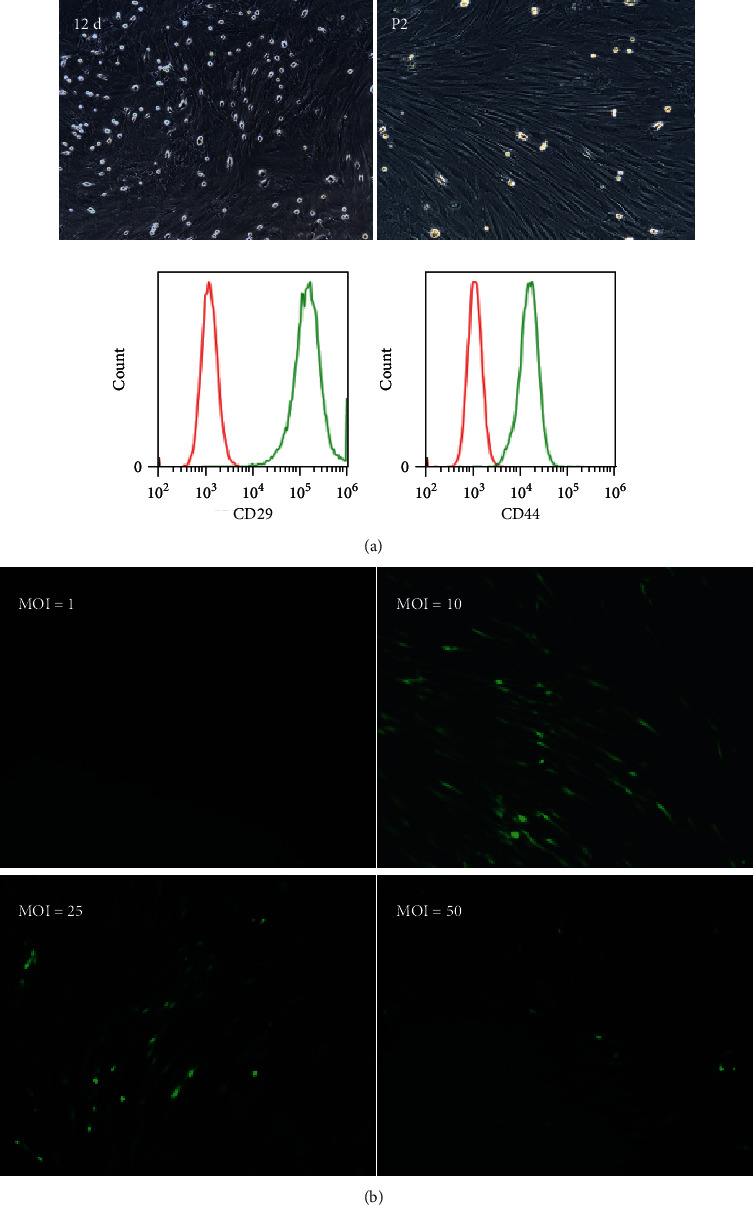
BMSC culture identification and Lv-BMP-7 transfection. (a) Morphological observation and identification of rat BMSCs (100x). Flow cytometry was used to detect CD29 and CD44 on the cell surface. The red peak was a negative control, and the green peak was a positive expression peak. (b) Lv-BMP-7 transfection efficiency identification with different MOIs (100x).

The growth of rat BMSCs was observed under an inverted phase-contrast microscope. Our results showed that, at Day 12, the cells were tightly arranged, with the polygonal and/or fusiform cell morphology. For the secondary passage of BMSCs, the cells showed a fusiform shape, which aggregated and grew in the vortex-like pattern, also tightly arranged. Moreover, the cells were rich in cytoplasm, with large nuclei and clear nucleoli, showing the typical shape of BMSCs (Figure 1(a)). Flow cytometry indicated the expression of CD29 and CD44 markers on the surface of P2 BMSCs. The results showed that the positive expression rate of CD29 on the surface of P2 BMSCs was 99.44%, and the positive expression rate of CD44 was 99.17% (Figure 1(a)). Moreover, the fluorescence inverted microscope was used to detect the transfection efficiency of LV-BMP-7 with different MOI values (TU/ml). Our results showed that, after 72 h of transfection, no GFP-positive cells were observed in the MOI = 1 group, while GFP-positive cells were observed in the MOI = 10, 25, and 50 groups. Among these groups, the fluorescence intensity of the MOI = 10 group (12.40 ± 0.07) was significantly higher than the MOI = 25 (2.79 ± 0.05) and MOI = 50 (0.83 ± 0.02) groups (both *P* < 0.05) (Figure 1(b)). These results suggest that MOI = 10 is the optimum multiplicity of infection for Lv-BMP-7.

### 3.2. Analysis of Cell Viability and ALP Activity

**Figure 2:**
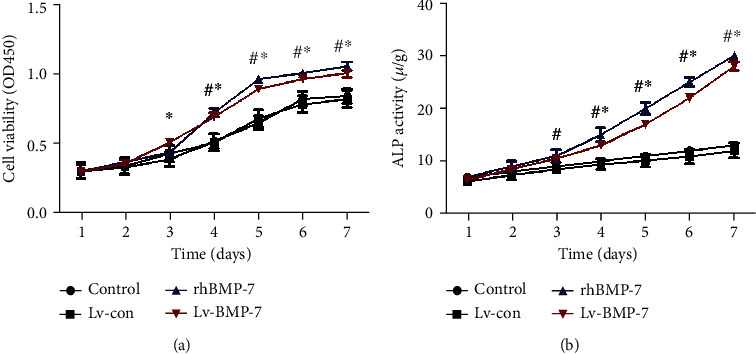
BMSC cell viability and ALP activity assessment. (a) BMSC cell viability assessment. (b) ALP activity assessment. Compared with the control and lentiviral vector control groups, ^∗^*P* < 0.05.

The cell viabilities at different time points were assessed with the CCK-8 assay. Our results showed that there was no significant difference in the cell viabilities between the control and lentiviral vector control groups (*P* > 0.05). For the rhBMP-7 group, starting from Day 4 after intervention, the cell survival rate was significantly higher than the control group (*P* < 0.05). For the Lv-BMP-7 group, starting from Day 3 after intervention, the cell survival rate was significantly higher than the lentiviral vector control group (*P* < 0.05). No significant differences were observed in the cell viability between the rhBMP-7 and Lv-BMP-7 groups (*P* > 0.05). Similar results were observed for the ALP activity as the findings from the CCK-8 assay (Figure 2). These results suggest that both rhBMP-7 and Lv-BMP-7 can promote the proliferation and differentiation of BMSCs.

### 3.3. Morphological Differentiation of BMSCs into Neuron-Like Cells

**Figure 3:**
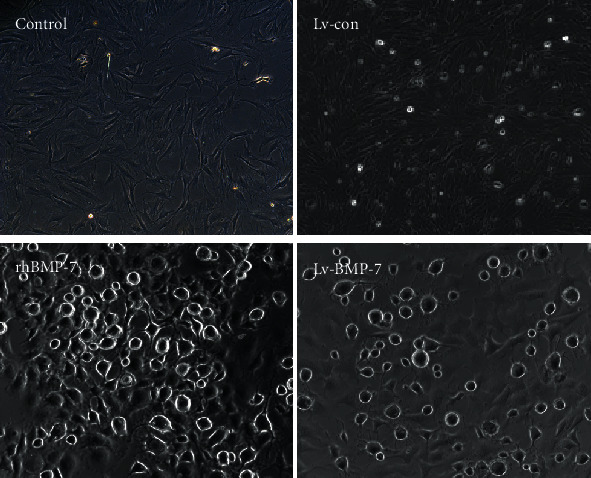
Morphological changes of BMSCs. After intervening with rhBMP-7 and Lv-BMP-7, the morphological changes of BMSCs were observed (200x).

After intervention with rhBMP-7 (75 ng/ml) for 2 h, the morphology of BMSCs was observed. Our results showed that some BMSCs had enhanced light refraction, with protrusions (which had secondary branches, just like neuronal cells in morphology). After 72 h of transfection with Lv-BMP-7, the morphologies of BMSCs changed significantly. The cell body contracted into circles, with cellular protrusions, exhibiting typical neuron-like morphologies (Figure 3). These results suggest that both the rhBMP-7 and the Lv-BMP-7 could induce BMSCs to differentiate into neuron-like cells.

### 3.4. Cellular Immunofluorescence of Neuronal Cell Marker Expression

**Figure 4:**
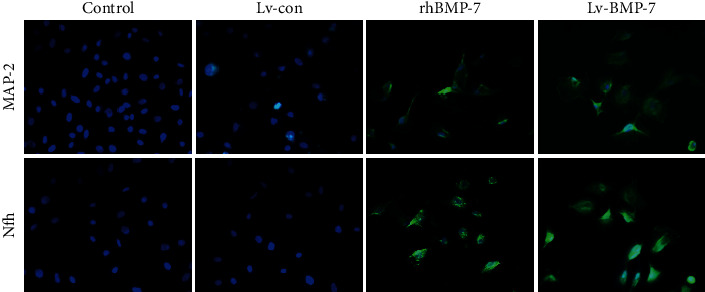
Cell immunofluorescence detection. The expression and location of MAP-2 and Nfh were detected with the cell immunofluorescence (200x).

The expression levels of neuronal markers MAP-2 and Nfh were then detected with the cellular immunofluorescence. Our results showed that, after the intervention of rhBMP-7 and Lv-BMP-7, both the MAP-2 and the Nfh were positively expressed in the neuron-like cells differentiated from BMSCs. The MAP-2 was mainly distributed within the cell body and processes, while the Nfh was mainly distributed within the cytoplasm and processes (Figure 4).

### 3.5. The mRNA Expression Levels of Neuronal Markers MAP2 and Nfh

**Figure 5:**
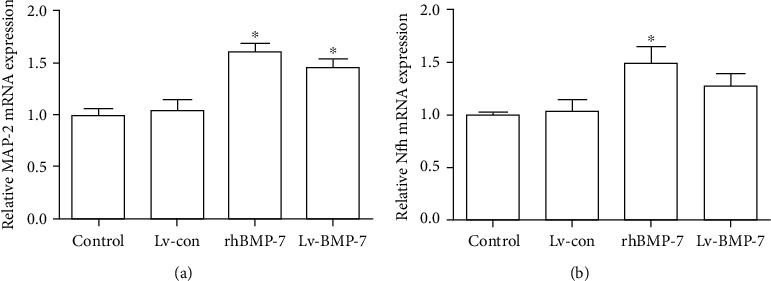
Relative mRNA expression levels of MAP-2 and Nfh. (a, b) Quantitative real-time PCR was performed to detect the mRNA expression levels of MAP-2 (a) and Nfh (b). Compared with the control and lentiviral vector control groups, ^∗^*P* < 0.05.

To investigate the mRNA expression levels of MAP2 and Nfh, the BMSCs were induced with rhBMP-7 (75 ng/ml) or Lv-BMP-7, and the quantitative real-time PCR was performed. Our results showed that there was no significant difference in the mRNA expression levels of MAP-2 between the control and lentiviral vector control groups (*P* > 0.05). Moreover, the mRNA expression levels of MAP-2 in the rhBMP-7 and Lv-BMP-7 groups were significantly elevated compared with the control and lentiviral vector control groups (*P* < 0.05), while no significant difference was observed between the rhBMP-7 and Lv-BMP-7 groups (*P* > 0.05) (Figure 5(a)). On the other hand, there was no significant difference in the Nfh mRNA expression level between the control, lentiviral vector control, and Lv-BMP-7 groups (*P* > 0.05). However, the mRNA expression levels of Nfh in the rhBMP-7 group were significantly higher than those in the control and lentiviral vector control groups (*P* < 0.05) (Figure 5(b)). These results suggest that the rhBMP-7 and Lv-BMP-7 induction would elevate the mRNA expression levels of neuronal markers MAP-2 and Nfh in the neuron-like cells.

### 3.6. Protein Expression Levels of Neuronal Markers MAP2 and Nfh

**Figure 6:**
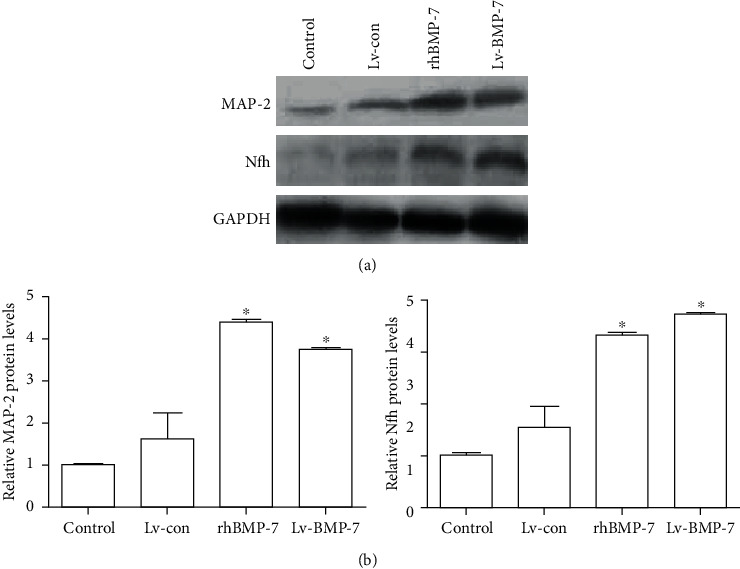
Relative protein expression levels of MAP-2 and Nfh. (a) Western blot analysis was performed to detect the protein expression levels of MAP-2 and Nfh. (b) Statistical analysis. Compared with the control and lentiviral vector control groups, ^∗^*P* < 0.05.

To investigate the protein expression levels of MAP2 and Nfh, the BMSCs were induced with rhBMP-7 (75 ng/ml) or Lv-BMP-7, and the Western blot analysis was performed. Our results showed that there was no significant difference in the protein expression levels of MAP-2 between the control and lentiviral vector control groups (*P* > 0.05). Moreover, the protein expression levels of MAP-2 in the rhBMP-7 and Lv-BMP-7 groups were significantly elevated compared with the control and lentiviral vector control groups (*P* < 0.05), while no significant difference was observed between the rhBMP-7 and Lv-BMP-7 groups (*P* > 0.05) (Figure 6(a)). On the other hand, there was no significant difference in the Nfh protein expression level between the control and lentiviral vector control groups (*P* > 0.05). However, the protein expression levels of Nfh in the rhBMP-7 and Lv-BMP-7 groups were significantly higher than the control and lentiviral vector control groups (*P* < 0.05) (Figure 6(b)), while no significant difference was observed between the rhBMP-7 and Lv-BMP-7 groups (*P* > 0.05) (Figure 6(b)). Taken together, these results suggest that the rhBMP-7 and Lv-BMP-7 induction would elevate the protein expression levels of neuronal markers MAP-2 and Nfh in the neuron-like cells.

## 4. Discussion

Transplantation of MSCs after SCI has currently been a hot spot for the SCI treatment. BMSCs have the potential to differentiate into nerve cells, with easy access and no ethical controversy. At present, BMSCs have been recognized as ideal seed cells for the stem cell transplantation to treat SCI. BMP belongs to the TGF-*β* superfamily (except for BMP-1). BMP-7 is one of the important members of the BMP family. In addition to its osteogenic regeneration ability, BMP-7 has been shown to act as a trophic factor in all the regeneration processes of cartilage, tendon, myogenesis, and bones, and it could promote the differentiation, proliferation, and synaptic regeneration of nerve cells [17–21]. Our previous study has shown that the spinal arachnoid injection of BMP-7 would promote neuronal regeneration and motor function recovery after acute SCI [16]. Lein et al. [10] have confirmed that BMP can induce nerve cells to exhibit the characteristics of stem cells. However, the role of BMP-7 on BMSCs has been poorly understood. Therefore, whether BMP-7 could induce BMSCs to differentiate into neuronal cells was investigated, to explore new ways of transplanting BMSCs to treat SCI.

Our results showed that, after the rhBMP-7 and Lv-BMP-7 induction, some of the rat BMSCs had changed morphologies, with increased cell body refraction. The cells had protrusions, and the morphology was very similar to neuronal cells. Moreover, the expression and localization of neuronal markers MAP-2 and Nfh in these induced neuron-like cells were detected. Nfh is mainly distributed in the cytoplasm and axons of neurons, which plays an important role in maintaining the morphology of neurons. Moreover, it also plays an important role in the axonal transport, brain development, neuron regeneration, and plasticity [22].

MAP2 is mainly distributed in the cell body of neurons, which can promote the formation of neuronal processes and contributing to maintain the stability of neuronal structures [23]. Our results from the quantitative real-time PCR, Western blot analysis, and immunofluorescence showed that the expression levels of MAP-2 and Nfh were significantly elevated in the BMP-7-induced BMSCs, suggesting that BMP-7-induced BMSCs not only had changed in morphology but also had changed on the molecular level. Guan et al. [24] have confirmed that BMP-7 can improve the nerve function of cerebral ischemic rats. Moreover, Perron and Dodd [25] have found that BMP-7 can promote neurite outgrowth. In the repairing process after the peripheral nervous system injury, the use of BMP will cause nerve dense morphology [26, 27]. The above studies have shown that BMP-7 plays an important regulatory role in the development and repairing process of the nervous system, thus exerting a neuroprotective effect. Meanwhile, the increased expression of MAP-2 and Nfh induced by BMP-7 could further promote the differentiation, proliferation, and synaptic regeneration of nerve cells. Our results also confirmed that both rhBMP-7 and Lv-BMP-7 induction promoted the proliferation and ALP activity of neuron-like cells induced by BMSCs, in line with previous findings from de Rivero Vaccari et al. [28].

In conclusion, our results showed that BMP-7 induced BMSCs to differentiate into neuron-like cells highly expressing neuronal cell markers. These findings provide basic evidence for the development of new treatment strategies for SCI. However, it is still not clear whether these cells had the function of interneural signal transduction and its therapeutic effect on SCI needed to be further confirmed in the future.

## Data Availability

The data used to support the findings of this study are available from the corresponding author upon reasonable request.
